# Comparing the Incidence of Buccal Mucosa Cancer in South Asian, White, and Black Populations Residing in the United States: A Cross-Sectional Analysis

**DOI:** 10.31557/APJCP.2021.22.1.195

**Published:** 2021-01

**Authors:** Stephen J Sozio, Sachin Jhawar, Yaqun Wang, Mutlay Sayan, Rahul Parikh, Sung Kim

**Affiliations:** 1 *Rutgers Cancer Institute of New Jersey, Department of Radiation Oncology, New Brunswick, NJ, USA. *; 2 *The Ohio State University Comprehensive Cancer Center, Columbus, OH, USA. *; 3 *Rutgers School of Public Health, Department of Biostatistics and Epidemiology, Piscataway, NJ, USA. *

**Keywords:** Buccal mucosa, cancer, incidence, Betel nut, Asian

## Abstract

**Background:**

Recreational use of the betel nut, which is common among the South Asian population, is a known risk factor for developing Head and Neck cancer. As South Asians comprise a significant proportion of the United States population, we seek to determine if those living within the country experience a higher rate of head and neck cancers compared to other races.

**Methods:**

Data of patients diagnosed with head & neck cancers from 2010-2016 was collected from the National Cancer Database^®^ and compared to race-matched US census data for each corresponding year to calculate incidence. Pairwise comparisons were performed between the incidence for South Asians versus Whites and South Asians versus Blacks using one sided Chi-square tests.

**Results:**

South Asians experienced a significantly higher incidence of buccal mucosa/vestibule cancers when compared to Whites or Blacks for every year between 2010-2016, but a comparatively lower incidence of larynx or oropharynx cancers.

**Conclusions:**

South Asians residing within the United States have a higher incidence of buccal mucosa/vestibule cancers, but a lower incidence of more common cancers, such as larynx or oropharynx cancer. This may suggest that the etiology behind the high buccal mucosa/vestibule cancer incidence is due to a social habit, as opposed to an inherent racial susceptibility.

## Introduction

Areca nut, commonly referred to as Betel nut, is the seed of the Areca catechu palm tree, which is indigenous to Southeast Asia, Pakistan, and India (Staples and Bevacqua, 2006). Betel nut is frequently consumed in the form of “Paan” which commonly contains a mixture of tobacco, seeds, quenched lime, spices, and areca nut enfolded in betel leaf. Paan is typically chewed and subsequently expectorated, and is perceived to have benefits like freshening the mouth, aiding digestion, germ-killing, astringency, mood enhancement, tension relief and oral cleaning. Gutkha is a commercially available form of Paan that is sweet and considered by children to be a form of candy (Niaz et al., 2017). In addition, chewing Betel nut is also known to result in some degree of psychostimulation. It is estimated that approximately 600 million people throughout the world regularly use betel nut, with multiple epidemiological studies projecting that between 3.3 to 37% of the Indian and Pakistani populations routinely use this substance (Niaz et al., 2017; Bedi and Scully, 2014). Betel nut is estimated to be the fourth most common addiction in the world (Bedi and Scully, 2014).

Multiple studies associate the longitudinal use of Betel nut (with or without the concurrent use of tobacco) with oral cavity and buccal mucosa squamous cell carcinoma (SCC) (Niaz et al., 2017; Bedi and Scully, 2014; Warnakulasuriya et al., 2002; Stich et al., 1982; Dave et al., 1992). Its composition of significant levels of tannins and alkaloids can result in cytotoxicity and genotoxicity, leading to malignant transformation of surrounding tissue (Bedi and Scully, 2014). This is in line with the disproportionally higher recorded incidences and observed mortality of oral cavity and buccal mucosa SCC in South Asia (Pakistan and India) compared to the remainder of the world, with current age-standardized incidence rates estimated to be 12.2/100,000 and 9.1/100,000, respectfully (Warnakulasuriya et al., 2002; Bray et al., 2018). Despite the widely available literature detailing the increased incidence of oral cavity/buccal mucosa cancers in India and Pakistan, little has been published regarding the important question of whether this increased incidence also applies for South Asians residing in the United States.

The yearly incidence of oral and pharyngeal cancers in the United States is about 30,000, with buccal mucosa SCC making up about 10% of the oral cavity cases (Silverman, 2001; Dyalram, 2017). The disproportionate incidence of buccal mucosa cancer in India and Pakistan naturally leads to the question of whether this is a health problem among South Asians residing in the United States also. Our hypothesis is that South Asians in the United States have a higher incidence of cancers of the buccal mucosa/vestibule compared to Whites and Blacks (Bonkowsky and Gerdemann, 2019). Because we strongly suspected that any increased incidence would be due to social habits as opposed to any inherent racial susceptibility, we also looked at the incidence rates by race for more common head & cancers, namely oral cavity, oropharynx and larynx cancer- if South Asian incidence rates were high for buccal mucosa cancer but not for more common cancers, that would be highly suggestive that a social habit was responsible.

## Materials and Methods

To establish a representative sample of patients suffering buccal mucosa, oral cavity, oropharynx, and larynx SCC within the United States, our group utilized data available within the National Cancer Database® (NCDB), a comprehensive database jointly sponsored by the American Cancer Society and the American College of Surgeons. This database features anonymized data of patients diagnosed with cancer from 2004-2016, collected from hospital registry databases at over 1,500 Commission on Cancer accredited facilities within the United States, which is estimated to capture over 70% of newly diagnosed cancers within the country yearly (Bilimoria et al., 2008). 

Initially, the raw data of patients diagnosed with buccal mucosa, oral cavity, oropharynx, and larynx SCC was obtained from NCDB and subsequently filtered to only include patients of White, Black, or South Asian (Indian/Pakistani) race. Next, the filtered dataset was sorted based on location of the patient’s primary cancer as determined by their primary diagnosis ICD-10 code, as illustrated in Supplemental Table 1. Buccal mucosa/vestibule are often considered to be subsites of oral cavity, but for the purposes of this study, were kept separate. The reasoning is that betel nut is frequently held between the cheek and gum before being either expectorated or ingested, so there is likely more direct contact between the carcinogens and the buccal mucosa and vestibule (space between the lips/cheeks and teeth/gums) than there is direct contract with the oral cavity proper (oral tongue, floor of mouth, hard palate).

When calculating yearly incidence rates, the positive cancer cases were pulled from filtered NCDB data, and the associated denominator by race for each year (supplement Table 2) was accessed from the United States census bureau’s platform (data.census.gov). As readily searchable data on the US Census platform was only available from the year 2010 and later, our cancer dataset was subsequently filtered to only include patients diagnosed with cancer between 2010-2016 to ensure an appropriate denominator was available to match with the numerator. The population of White, Black, South Asian (Indian/Pakistani) populations within the United States was obtained using American Community Survey 1-Year Estimate reports, which feature data collected yearly from 2010-2016 (Table ID: B02001, B02015). As NDCB data only encompasses approximately 70% of new cancer diagnoses in the country, each population statistic was then multiplied by 0.7 to obtain an adjusted estimated population density.

Each race cohort within the cancer dataset was sorted by year of diagnosis and type of head & neck cancer diagnosis, and subsequently matched to its corresponding adjusted population density, as calculated above. Incidence was then calculated by dividing these two numbers. Pairwise comparisons were performed between the incidence for South Asians vs Whites and South Asians vs Blacks using one sided Chi-square tests. P-value < 0.05 was considered as significant. R (www.r-project.org) was used to conduct the analyses.

**Table 1:** Cases of Head and Neck Cancer Diagnosed from 2010-2016 Sorted by Primary Site and Race

	Buccal mucosa/vestibule	Oral cavity	Larynx	Oropharynx
South Asian	316	442	183	195
White	12,494	41,937	56,885	84,654
Black	883	3,263	9,706	8,168

**Figure 1:**
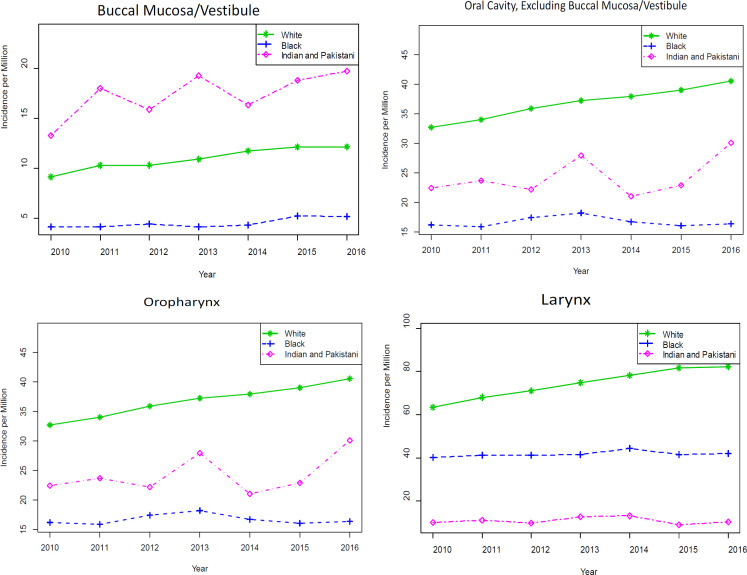
Graphical Representation of Yearly Estimated Incidence Per Million Population of Buccal Mucosa/Vestibule, Oral Cavity (excluding Buccal Mucosa/Vestibule), Oropharynx, and Larynx Cancers Diagnosed in White, Black, and South Asian Populations Living within the United States between 2010-2016

**Table 2:** Yearly Estimated Incidence Per Million Population of Buccal Mucosa/Vestibule, Oral Cavity, Oropharynx, and Larynx cancers by Race & Pairwise Comparisons

Year of Diagnosis	Incidence in South Asian Population	Incidence in White Population	Incidence in Black Population	p-value South Asian vs White	p-value South Asian vs Black
Buccal Mucosa/Vestibule					
2010	13.27	9.14	4.15	0.0294	4.73E-09
2011	17.97	10.28	4.12	0.000254	1.99E-18
2012	15.89	10.31	4.43	0.00531	2.83E-13
2013	19.27	10.91	4.15	5.12E-05	6.71E-23
2014	16.31	11.74	4.28	0.0178	2.60E-16
2015	18.79	12.13	5.23	0.000843	6.34E-18
2016	19.72	12.14	5.17	0.000144	1.23E-20
Oral Cavity, excluding Buccal Mucosa/Vestibule					
2010	22.42	32.7	16.17	0.00486	0.0181
2011	23.67	34.01	15.86	0.00455	0.00336
2012	22.17	35.89	17.41	0.000259	0.0557
2013	27.93	37.24	18.18	0.00896	0.00042
2014	21.02	37.94	16.7	3.51E-06	0.0571
2015	22.88	39.01	16.04	6.90E-06	0.00398
2016	30.08	40.55	16.35	0.00272	5.54E-08
Oropharynx					
2010	10.07	63.42	40.2	3.39E-23	2.60E-12
2011	10.96	68.03	41.3	1.25E-25	1.29E-12
2012	9.62	71.02	41.25	1.69E-29	3.58E-14
2013	12.59	74.78	41.48	1.74E-30	1.25E-12
2014	13.04	78.14	44.47	1.92E-34	8.57E-15
2015	8.88	81.67	41.46	3.05E-43	8.37E-18
2016	10.36	82.3	41.92	7.84E-43	6.40E-17
Larynx					
2010	8.24	50.48	51.23	1.25E-18	8.75E-19
2011	10.08	49.53	50.56	2.11E-17	1.14E-17
2012	8.36	49.98	49.14	7.53E-20	3.36E-19
2013	11.01	50.69	48.24	5.44E-19	2.05E-17
2014	10.15	49.87	50.88	7.98E-21	4.40E-21
2015	10.59	49.99	50.76	1.32E-21	1.03E-21
2016	11.7	48.74	46.46	3.95E-20	2.33E-18

## Results

Table 1 reveals that the number of cases of head & neck cancers by primary site is starkly different in South Asians versus Whites and Blacks for the entire time period of 2010-2016. In Whites and Blacks, the number of buccal mucosa/vestibule cancers (and oral cavity cancers to a lesser degree) is far exceeded by incidence of larynx and oropharynx cancers, whereas in South Asians, the number of buccal mucosa/vestibule cancers is actually higher than for larynx or oropharynx cancers. 

For every year between 2010 and 2016, there was a significantly higher incidence of buccal mucosa/vestibule cancer diagnosed in the South Asian population compared to the White and Black population (Table 2 and Figure 1). The same is not true for any of the other, more common head and neck cancers, as demonstrated in Table 2 and Figure 1. South Asians had a lower incidence of both oropharynx and larynx cancer than both Whites and Blacks. South Asians had a lower incidence of oral cavity cancers than Whites and a somewhat similar incidence to Blacks.

## Discussion

The literature consistently demonstrates that the longitudinal use of Betel nut (with or without the concurrent use of tobacco) is associated with the disproportionately increased risk of cancer of the buccal mucosa in India and Pakistan. Our hypothesis was that South Asians living in the United States might also have a higher incidence of buccal mucosa/vestibule cancers compared with Whites and Blacks. Because these are relatively rare tumors, we used a national representative cohort database (NCDB) to perform our analysis. 

Our results demonstrate that from 2010-2016, the incidence of buccal mucosa/vestibule cancer among South Asians living in the US significantly exceeded that of Whites and Blacks for every year. Furthermore, when looking at more common cancers, South Asians had significantly lower incidence of oropharynx and larynx cancer than both Whites and Blacks, and less oral cavity cancer than Whites. Because this is a national database study, drawing definitive conclusions regarding the reason for these differences is difficult, but the fact that South Asians had a significantly increased incidence in this one, relatively rare primary site, appears to support a social habit etiology as opposed to an inherent racial susceptibility. The literature from India and Pakistan would strongly suggest that this social habit is the use of betel nut products. 

While there are a number of risk factors for developing buccal mucosa cancer, the most common is the routine use of the betel nut and smoking (Kumar et al., 2016). Betel nut use is highly prevalent among the Asian Indian and Pakistani populations, but largely uncommon within the White and Black populations of the United States (Sharan et al., 2012). While there is little literature directly exploring the epidemiology and frequency of Betel nut use within the United States, the product is inexpensive and readily available throughout the country, often sold in specialty grocery stores or through online vendors (Blank et al., 2008). When these factors are viewed in the context of the relatively rare overall prevalence of buccal mucosa SCC within the United States, the disproportionally higher incidence within the South Asian Indian population holds significant importance, and is possibly attributed to the use of this product within these communities (Silverman, 2001; Dyalram, 2017). 

Interestingly, in the current study, the incidence of oral cavity cancers (as defined in this paper) within the South Asian populations was significantly lower than that of the White population. Note that in this study, we differentiated between buccal mucosa/vestibule and oral cavity, whereas the oral cavity is usually defined inclusive of the buccal mucosa/vestibule. Here, the oral cavity was defined as including the most common oral cavity subsites, namely the tongue, floor of mouth and hard palate. If the increased incidence of buccal mucosa/vestibule cancers is due to betel nut consumption, one may question why this does not appear to result in an increased overall incidence of oral cavity cancers in the South Asian population. We suspect that this may be because the Betel nut is typically held in the mouth within the space between the cheek and the teeth before being spit out or ingested, so there may be more direct contact with the cheek and vestibule as opposed to the tongue, floor of mouth or hard palate. 

While tobacco is often added to the Paan mixture for increased psychostimulant effect, tobacco use cannot explain the increased incidence of buccal mucosa/vestibule cancers in the South Asian community. Current projections from the Center for Disease Control estimate that South Asians actually use tobacco products less often compared to Whites and Blacks (10% of South Asians vs 20-23% of Whites and Blacks) (Schoenborn et al., 2013; Centers for Disease Control and Prevention, 2014). Furthermore, multiple studies have documented that the use of Betel nut with or without tobacco is associated with development of buccal mucosa neoplasm (Sharan et al., 2012; Van Wyk et al., 1993; Auluck et al., 2009). 

This study would seem to indicate that specific patient-centric educational forums are necessary to reduce the risk of buccal mucosa cancer in South Asians living in the US. The incidence of this disease in South Asians is disproportionately high, and appears to be increasing over time. Though the existing literature would seem very clear regarding the dangers of betel nut use, this practice remains common among South Asians, reflective perhaps of the religious and cultural importance of betel nut products, especially in the Hindu population. In India, oral cancer is more prevalent among Hindus than Muslims, with Christians having the least prevalence, indicating perhaps that each South Asian religious group has its own risk of oral cavity cancer in the US, and needs to be reached in an individual manner about the dangers of betel nut use. 

This study does have some of the typical drawbacks associated with hospital-based registry studies, the most significant being the lack of detailed information on patients’ social habits ie who smoked tobacco products, used chewing tobacco, or used betel nut. Without knowledge of independent risk factors such as the smoking history of patients included in this study, it is difficult to conclude that betel nut consumption alone led to the increased incidence of buccal mucosa/vestibule cancers among US South Asians. In addition, as NCDB only accounts for 70% of cancers diagnosed in the country, the remaining proportion of cancers diagnosed within the United States were not included within this analysis. Given the restrictions associated with this study, additional studies correlating Betel nut use and buccal mucosa/vestibule cancer should be considered.
